# Seroprevalence of neutralizing antibodies against human adenovirus type 55 in the South Korean military, 2018-2019

**DOI:** 10.1371/journal.pone.0236040

**Published:** 2020-07-16

**Authors:** So Yun Park, Jae-Hoon Ko, Sezim Monoldorova, Jonguk Jeong, Bo-Young Jeon, Soon-Hwan Kwon

**Affiliations:** 1 Department of Infectious Diseases, Armed Forces Medical Research Institute, Daejeon, Republic of Korea; 2 Division of Infectious Diseases, Department of Medicine, Samsung Medical Center, Sungkyunkwan University School of Medicine, Seoul, Republic of Korea; 3 Department of Biomedical Laboratory Science, College of Health Science, Yonsei University, Wonju, Republic of Korea; Cincinnati Children's Hospital Medical Center, UNITED STATES

## Abstract

We conducted a seroprevalence study of a large ongoing outbreak of human adenovirus type 55 (HAdV-55) among the military in South Korea. Serum samples were collected between 2018 and 2019 from military-exposed (military group) and non-exposed (non-military group) populations. The plaque reduction neutralization test (PRNT) was used to assess neutralization activity against HAdV-55. A total of 100 sera was collected from the non-military group, of which 18.8% showed HAdV-55 neutralizing antibody activity. Ninety-six sera were tested from the military group, which had significantly higher prevalence of neutralizing antibodies (56.0%, *P* <0.001). A significantly higher proportion of the military group had PRNT titers ≥1:1,000 than the non-military group (85.7% vs. 50.0%, *P* = 0.004). Among the military group, 48.9% of active-duty soldiers had PRNT titers ≥1:5,000, while none of the discharged civilians did (*P* = 0.007). In conclusion, Koreans were exposed to HAdV-55 in their communities, but the exposure risk was higher among people in military service.

## Introduction

Human adenovirus type 55 (HAdV-55) is an emerging recombinant HAdV strain of types 11 and 14, causing a potentially fatal acute respiratory illness (ARI) in adult populations [1, 2]. Since the first Chinese outbreak of HAdV-55 in 2006 [3], sporadic severe cases and multiple outbreaks in civilian and military communities have been reported in China [1–5]. A Chinese seroprevalence study evaluating healthy blood donors in Guangzhou in 2015 reported seropositive rates for HAdV-55 at 22.4% with gradual increases associated with age, suggesting that HAdV-55 became a strain circulating among the Chinese community [6].

In South Korea, HAdV-55 has been detected among severe pneumonia patients in the military since 2012 [7–9]. In the winter of 2014, HAdV-55 caused a large ongoing outbreak within the military and is currently considered a major pathogen of severe pneumonia among military personnel [10, 11]. However, a molecular typing study of respiratory HAdV in the Korean civilian community has not been conducted since 2010 [12], and the current epidemiologic status of HAdV-55 among Koreans who have been discharged from military service is unknown. In May 2017, the first HAdV-55 outbreak among the Korean civilian community was reported from a high school, emphasizing the need for an epidemiologic investigation of HAdV-55 in Korea [13]. Thus, we investigated seroprevalence and distribution of neutralizing antibodies (nAb) against HAdV-55 and HAdV-4 among healthy civilian volunteers and military personnel in various settings.

## Methods

### Study population and serum samples

To evaluate HAdV-55 seroprevalence in Korean society, we collected serum samples from four distinct populations: 1) healthy civilian volunteers, including male citizens discharged from obligatory military service; 2) new military recruits collected during the entrance medical examination; 3) active-duty soldiers admitted to a military hospital for ARI caused by respiratory viruses other than HAdV; and 4) active-duty soldiers after one year of service, collected during routine medical examinations.

In South Korea, male citizens are conscripted in their early 20s and serve for approximately two years. Because HAdV-55 was first detected in 2012 and then another military outbreak, which remains ongoing, followed two years later [8–10], individuals who entered military service after 2012 are more likely to have nAb against HAdV-55. To evaluate seroprevalence according to military exposure status, we categorized populations into those who had been exposed to the military environment since 2012 (military group) and those who had not (non-military group). Grouping details are presented in Table 1. This study was approved the Institutional Review Boards of the Armed Forces Medical Command (AFMC-18004-IRB-18-005) and Yonsei University (1041849-201808-BM-084-02). Written informed consent was obtained from each volunteer and patient.

**Table 1:** Study population characteristics according to military status.

Category	Study population, number	Serum collection date	Age, median (IQR)	Notes
**Non-military group**	New recruits, before initiation of military training, n = 24	July 2018	20 (20–22)	Serum samples were collected on the 2^nd^ day of military training.
	Healthy male civilian volunteers, without military service experience after 2012, n = 13	March-May 2019	22 (21–25)	Includes male citizens who were not yet conscripted, were waived for service, and who completed service before 2012.
	Healthy female civilian volunteers, n = 59	March-May 2019	21 (20–23)	Female citizens are not mandatorily conscripted for military service, and none of volunteers had served in the military as occupational soldiers.
**Military group**	Soldiers admitted to a military hospital for ARI, excluding HAdV infections, n = 31	March 2018-January 2019	21 (20–21)	All patients were tested with a multiplex respiratory virus panel and those infected with HAdV were excluded.
	Soldiers after a year of service, n = 50	May 2019	21 (21–22)	Serum samples were collected during routine medical examinations.
	Healthy male civilian volunteers, with military service experience after 2012, n = 19	March-May 2019	24 (23–25)	All completed their service after 2012.

Abbreviations: IQR, interquartile range.

### Laboratory procedures

The plaque reduction neutralization test (PRNT) was used to assess neutralization activity against HAdV-55 in the collected sera. All samples were aliquoted and frozen at –80°C after collection. Before the neutralizing assays, the serum samples were heat inactivated at 56°C for 30 min, and then stored at 4°C. Neutralization activity against HAdV-4, a community-circulating HAdV strain, was also evaluated as a control [12, 14]. We used a rabbit immunization model to establish PRNTs against HAdV-55 and HAdV-4 and confirmed the no cross-reactivity between HAdV-4 and HAdV-55 sera. HAdV-55 (NCCP43158) and HAdV-4 (NCCP43156) were distributed by the National Culture Collection for Pathogens in the Korean Centers for Disease Control and Prevention. A549 human lung carcinoma cells (ATCC, Manassas, VA, USA) were maintained at 37°C under 5% CO_2_ in Eagle's minimal essential medium (MEM) supplemented with 10% heat-inactivated fetal bovine serum and 1% penicillin-streptomycin. A549 cells were plated at a density of 7x10^5^ cells per well in 6-well plates. HAdVs (100 plaque-forming units (PFU) per well) were incubated for 1 h at 37°C either alone or together with serial dilutions of human sera or rabbit antisera. The positive control were used 2-fold dilution of the rabbit sera beginning with a 1:20 dilution (1:80; 1:160; 1:320; 1:640 and 1:1280), the human sera were used 5 or 10-fold dilution beginning with a 1:100 dilution (1:100; 1:500; 1:1000; 1:5000 and 1:10000). Subsequently, each sample was added to the 6 well plates and incubated at 37°C for 5 hr. The supernatant was then aspirated and overlaid with 3 mL per well of MEM with 2% heat-inactivated fetal bovine serum containing 0.8% SeaPlaque agarose (Takara). Following a 5 day incubation at 37°C, virus plaques were counted following crystal violet staining. nAb titers were determined by calculating the reciprocal of the serum dilution that inhibited 50% of the HAdV plaque compared with the negative control. To assess nAb presence, all collected sera were tested from a 1:20 dilution with a log2 dilution series, and those with a titer ≥1:80 were considered positive. Sera positive for HAdV-55 were titrated with 1:100, 1:500, 1:1000, 1:5000, and 1:10000 dilutions to assess the degree of neutralization activity.

### Statistical analyses

To compare prevalences among the different groups, either the chi-square or Fisher’s exact test was used. All *P*-values were two-tailed, and *P*<0.05 was considered statistically significant. IBM SPSS Statistics version 20.0 (IBM, Armonk, NY, USA) was used for all statistical analyses.

## Results

### Seroprevalence of HAdV-55 and HAdV-4, according to military status

**Fig 1:**
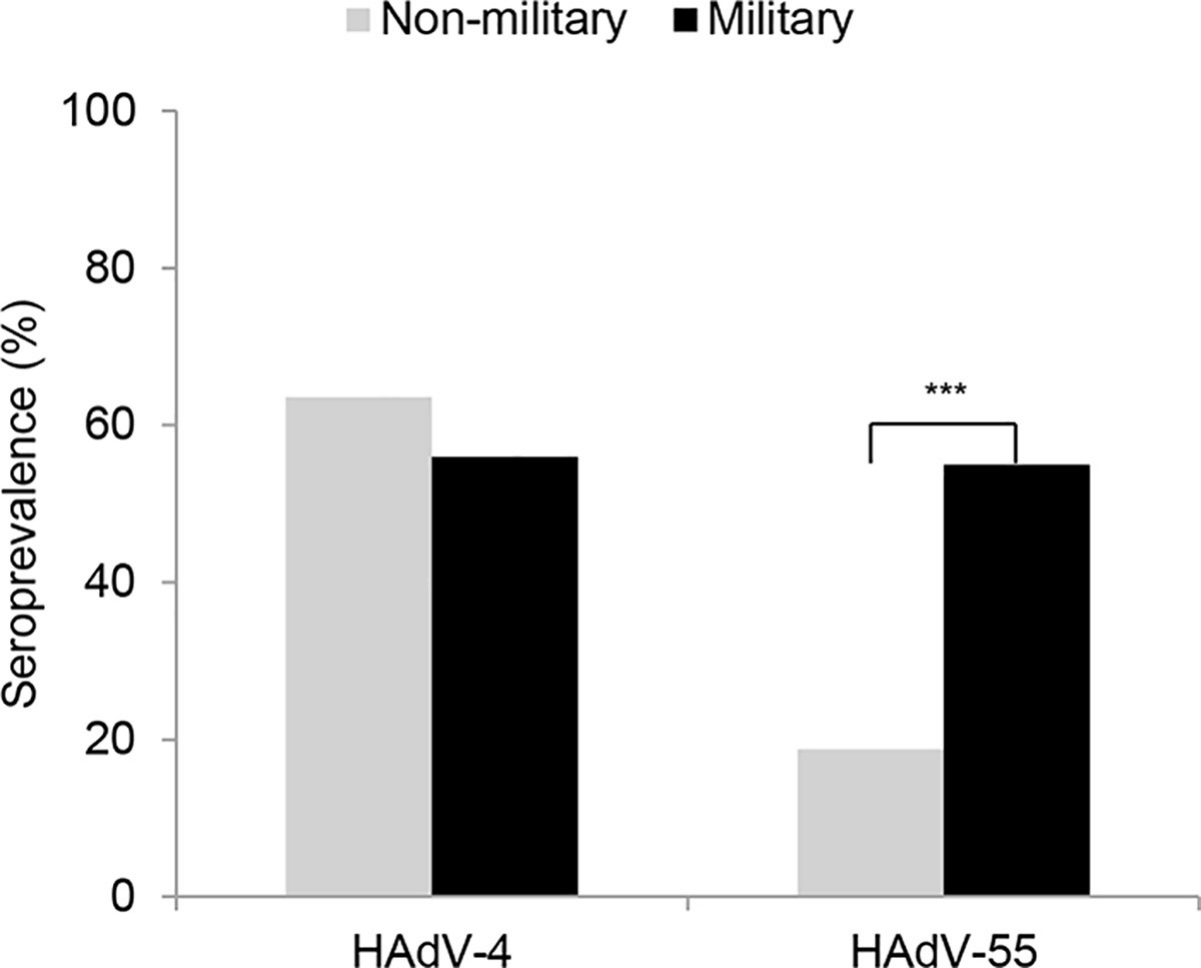
Seroprevalence of neutralization antibodies against HAdV-55 and HAdV-4 in Korean society according to recent military exposure. The data were analyzed with chi-square test. ***P<0.001.

A total of 196 sera was collected, and 74 (37.8%) were positive for HAdV-55 and 116 (59.2%) were positive for HAdV-4 (Table 2). Eighteen sera from the non-military group (18.8%) were positive for HAdV-55, and the positivity rate was significantly higher in the military group (56.0%, *P* <0.001). However, HAdV-4 seropositivity rates were not different between the non-military and military groups (63.5% and 55.5%, *P* = 0.247) (Fig 1). Similarly, HAdV-55 seropositivity rates were not different between the HAdV-4 positive and negative sera (65.3% and 62.0%, *P* = 0.296) (S1 Fig). The HAdV seropositivity rates among the sub-populations of both groups were not statistically different.

**Table 2:** Seroprevalence of neutralization antibodies against HAdV-55 and HAdV-4 in Korean society according to recent military exposure.

Non-military group			Military group		
Population	Positive/tested sera (%)		Population	Positive/tested sera (%)	
	HAdV-55	HAdV-4		HAdV-55	HAdV-4
New recruits, before training	5/24 (20.8)	14/24, (58.3)	ARI Soldiers, HAdV infection excluded	15/31 (48.4)	19/31 (61.3)
Male civilians, without recent military exposure	2/13 (15.4)	7/13 (53.8)	Soldiers, routine medical examination	32/50 (64.0)	25/50 (50.0)
Female civilians	11/59 (18.6)	40/59 (67.8)	Male civilians, military service after 2012	9/19 (47.4)	11/19 (57.9)
Total	18/96 (18.8)	61/96 (63.5)	Total	56/100 (56.0)	55/100 (55.0)

### PRNT titers against HAdV-55 among seropositive people

**Fig 2:**
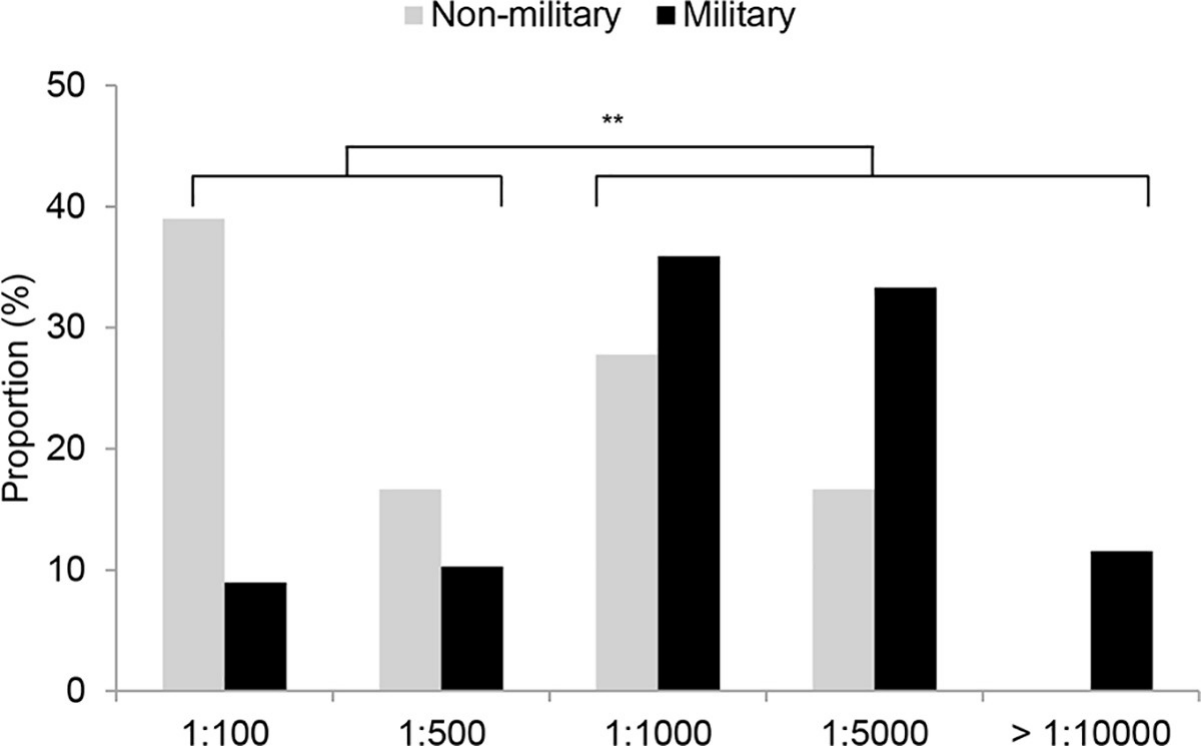
Distribution of neutralizing antibody titer against HAdV-55 according to military status. The data were analyzed with chi-square test. **P<0.01.

**Fig 3:**
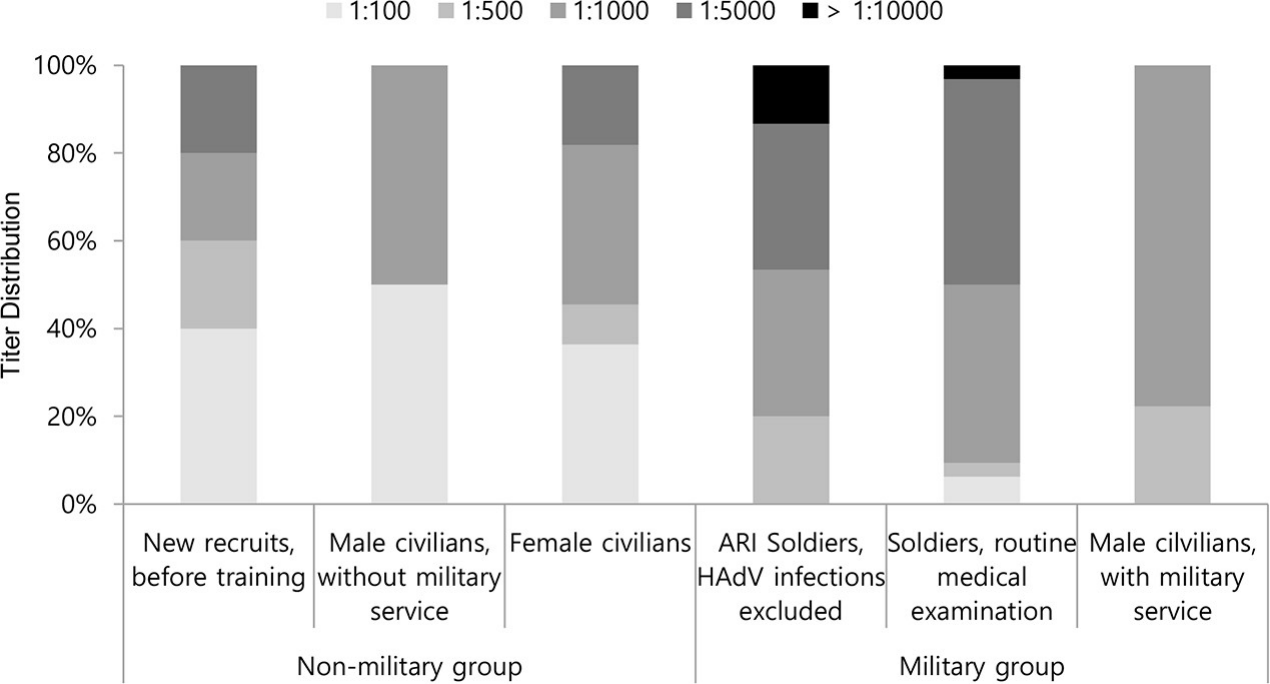
Titer distribution of neutralizing antibody against HAdV-55 according to population groups.

PRNT titration from 1:100 to 1:10,000 dilutions were conducted on the 74 HAdV-55-positive sera (Fig 2). Nine samples (12.2%) showed PRNT titers of 1:100, 8 (10.8%) showed titers of 1:500, 31 (41.9%) showed titers of 1:1000, 23 (31.1%) showed titers of 1:5000, and 3 (4.1%) showed titers ≥1:10000. The military group had a significantly higher proportion of PRNT titers ≥1:1000 (48 sera, 85.7%) compared with the non-military group (9 sera, 50.0%; *P* = 0.004). The distribution of PRNT titers in each sub-population is shown in Fig 3. Among the military group, 23 sera from active-duty soldiers (48.9%) showed PRNT titers ≥1:5000, while none of the discharged civilian sera had similar titers (*P* = 0.007). The distribution of PRNT titers was not different between the non-military subgroups. HAdV-55 titer distribution were not significantly different between the HAdV-4 positive and negative sera (S1 Fig).

## Discussion

Currently, the only epidemiologic investigation of HAdV-55 in a civilian community has been conducted in China, where several fatal HAdV-55 pneumonia cases have occurred among civilians [2, 6]. Several HAdV-55-associated ARIs have been reported in other countries, including France, Israel, and Singapore [15–17]. In South Korea, a military outbreak of HAdV-55 occurred in 2014 and remains ongoing [10, 11]. Because soldiers reside closely in barracks and contact with people outside the platoon is limited, the HAdV-55 outbreak was distinctly observed within the military population and it had a distinct epidemiologic pattern from that in the civilian community [11]. However, as approximately 300,000 Korean male citizens either enter the military or return to civilian society every year, transmission of HAdV-55 between the Korean military and civilian society is a great concern.

Seroprevalence of anti-HAdV-55 antibodies among the non-military exposed population was 18.8%. Although the kinetics of anti-HAdV-55 nAb have not been evaluated, the presence of PRNT titers ranging from 1:100 to 1:5,000 in this young population with a median age of 21 years suggests recent and remote exposure during adolescence. Molecular typing of respiratory HAdVs in Korea has not been reported since Lee *et al*., which reported 0.1% HAdV-55 prevalence among HAdV-positive throat swab samples collected in 2010 [12]. Our seroprevalence data imply that HAdV-55 spread widely into the Korean community after 2010. This is the first evidence that HAdV-55 became a community-circulating respiratory HAdV strain imported from China, and our results also suggest that a seroprevalence study or molecular typing of respiratory HAdVs is needed in neighboring countries and at-risk communities, including US military troops living in South Korea.

HAdV-55 seroprevalence was significantly higher in the military group compared with the non-military group, while HAdV-4 seroprevalence was not different between the two groups. More serum samples from the military groups had PRNT titers ≥1:1000 compared with the non-military group and more samples of active-duty soldiers had titers ≥1:5000 compared with discharged civilians. This finding is consistent with a previous HAdV-55 outbreak report and suggests that a proportion of the soldiers who participated in this study experienced a HAdV-55 infection during their military service. High PRNT titers ≥1:1000 in healthy blood donors were also observed in the Chinese seroprevalence study [6], where the researchers also found that strong nAb responses were more frequently observed against HAdV-55 than HAdV-14 and about half of HAdV-55-positive sera had titers ≥1:1000. By titrating sera up to a 1:10000 dilution, our study additionally showed that patients with recent HAdV-55 infections yielded nAb titers over 1:5000, which suggests that high titration is required for an antibody kinetics study of HAdV-55-infected patients.

In this study, most sera were collected from young people in their early 20s and we did not evaluate seroprevalence according to a range of age groups. However, if we did, we could have more accurately compared the serologic status of the non-military population with the military population, among whom a large HAdV-55 outbreak persists. Although approximately 100 samples were collected from the military and non-military groups each, the sample sizes for each sub-population were limited. However, by collecting serum samples from various populations, including both male and female civilians, and newly recruited, active-duty, and discharged soldiers, we were able to compare the effect of military exposure in greater detail.

In conclusion, seroprevalence of the non-military exposed population was 18.8%, suggesting that HAdV-55 has become a circulating respiratory HAdV strain among the Korean civilian community. Seroprevalence in the military population was also significantly higher than in the non-exposed sub-populations, with notably high titers (>1:5,000). Further epidemiologic studies across different age groups and neighboring communities are needed.

## Supporting information

S1 Fig Seroprevalence and titer of neutralization antibodies against HAdV-55 in HAdV-4 positive and negative sera. The data were analyzed with chi-square test.
(TIF)
