# Supplementary figures and images for: Seroprevalence of neutralizing antibodies against human adenovirus type 55 in the South Korean military, 2018-2019
Source: PLoS One. 2020 Jul 16;15(7):e0236040. doi: 10.1371/journal.pone.0236040 (PMC7365452; doi:10.1371/journal.pone.0236040)

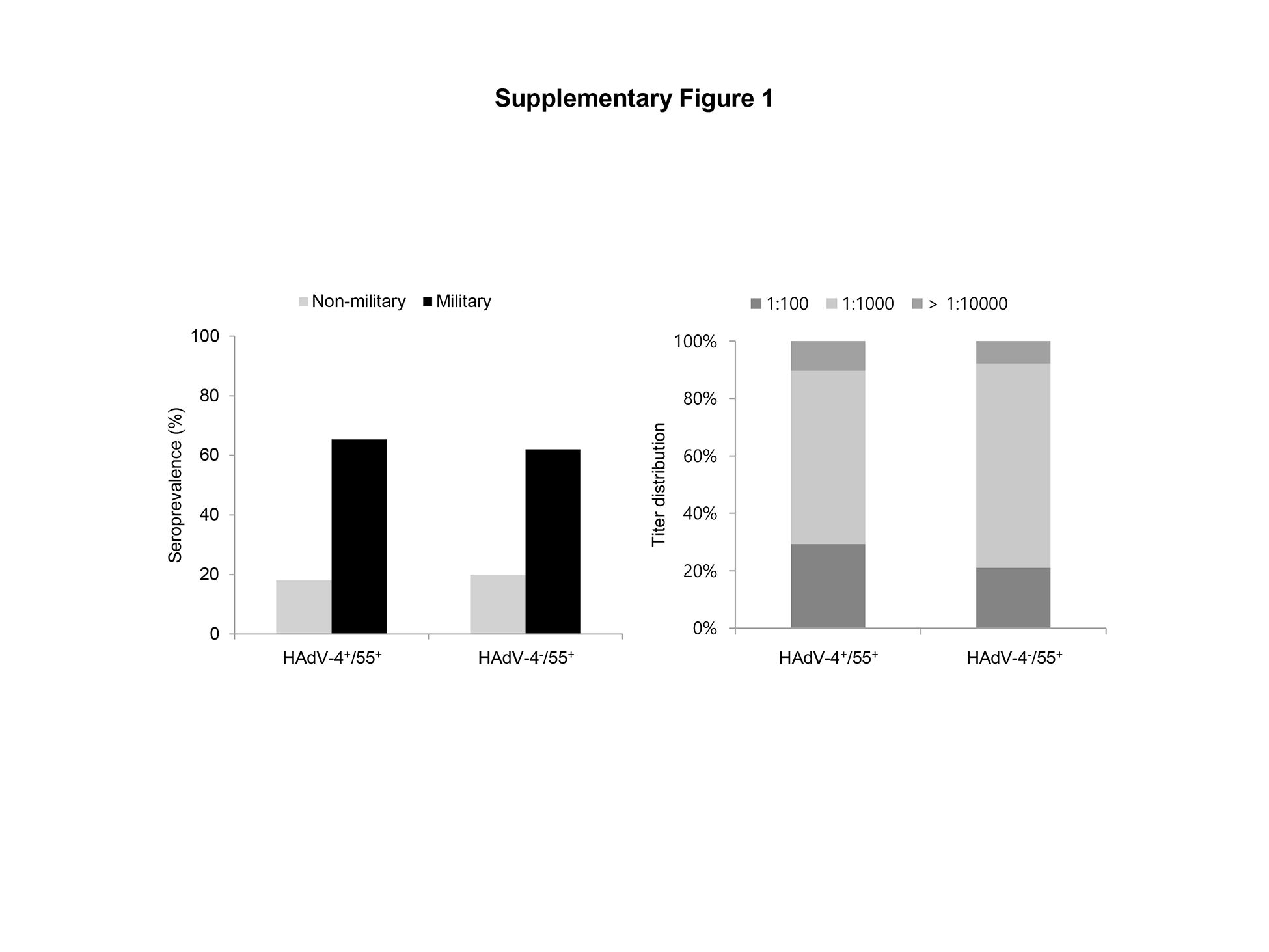

Supplement: S1 Fig — The data were analyzed with chi-square test. (TIF) [file pone.0236040.s001.tif]
